# Associations Between Hospital Structural Characteristics and Adoption of Public Health Data Integration and Automation: National Cross-Sectional proofsStudy

**DOI:** 10.2196/86263

**Published:** 2026-02-06

**Authors:** Hanadi Hamadi, Alaysia Alford, Chloe Smith Lopez, Durron Baker, James Ian Samaniego, Jessica Yu Jin

**Affiliations:** 1 Department of Health Administration, University of North Florida, 1 UNF Drive, Jacksonville, FL, 32224, United States, 1 9046205314

**Keywords:** public health data, data reporting, automation, active reporting, hospitals

## Abstract

**Background:**

Public health data integration and automation systems are crucial for effective health care delivery and public health surveillance. However, the factors associated with hospitals’ adoption and successful implementation remain inadequately explored.

**Objective:**

This study aims to examine how hospital characteristics influence the adoption of public health data integration and automation.

**Methods:**

We analyzed 2277 hospitals from the 2023 American Hospital Association Annual Survey and its Health Information Technology supplement, focusing on 6 public health reporting categories. Multivariable logistic regression models were used to examine the association between hospital characteristics and the 2 primary outcomes: active electronic data submission and use of automated transmission processes.

**Results:**

System-affiliated and not-for-profit hospitals demonstrated significantly higher rates of electronic data submission and automated reporting across most categories (odds ratio [OR] 1.70‐2.27; *P*<.001). Rural hospitals showed lower adoption rates in immunization registry (OR 0.77, 95% CI 0.61-0.97), public health registry (OR 0.67, 95% CI 0.46-0.97), and clinical data registry reporting (OR 0.77, 95% CI 0.60-0.98). Larger hospitals were more likely to implement electronic reporting, with medium and large hospitals showing stronger engagement in syndromic surveillance reporting (OR 1.52, 95% CI 1.06-2.19 and OR 2.29, 95% CI 1.17-4.46, respectively). Teaching status was significantly associated only with clinical data registry reporting (OR 2.66, 95% CI 1.56-4.52 for major teaching hospitals).

**Conclusions:**

Hospital characteristics, particularly system affiliation, ownership type, and geographic location, are strongly associated with public health data integration and automation capabilities. Findings suggest targeted interventions are needed to address disparities in smaller and rural facilities to ensure equitable advancement of public health reporting infrastructure.

## Introduction

The integration and automation of public health data have evolved from manual record-keeping to modern digital systems that enhance real-time data sharing and interoperability. Automated frameworks now combine structured and unstructured health data, improving research capabilities and public health responsiveness. The implementation of Findable, Accessible, Interoperable, and Reusable data principles has further enhanced data use for decision-making [1]. These innovations highlight the importance of technology-driven data integration in optimizing health care delivery and public health outcomes [2]. The Health Information Technology for Economic and Clinical Health Act of 2009, enacted into law by Title XIII of the American Recovery and Reinvestment Act of 2009, dramatically fueled computerization in health care through reimbursement incentives to adopt electronic health records (EHRs) as a method of standardizing and enhancing interoperability of data [3,4]. These differences reiterated that institutional resilience and organizational readiness were more critical than technology availability to successful adoption.

The 2020s have seen further advancements with artificial intelligence (AI) and machine learning technologies extending automation capabilities. AI-based software now streamlines tasks such as drug safety compliance reporting, reducing administrative burdens and human error [5]. Despite technological progress, persistent challenges remain: interoperability gaps prevent smooth data exchange between institutions due to diverse standards and proprietary tools [6]; regulatory requirements often fail to address structural barriers such as system upgrade costs or personnel training needs [5]; and workforce preparedness is frequently overlooked, particularly in low-resource settings where staff may lack proper training to use new technologies effectively [7].

Public reporting of hospital data, such as patient outcomes, infection rates, and readmission rates, can drive improvements in health care quality by promoting transparency and accountability. Studies have shown that hospitals participating in public reporting programs tend to engage in quality improvement activities more actively [8,9]. For instance, the American College of Cardiology’s voluntary public reporting program revealed that hospitals with higher participation rates demonstrated better performance in cardiac care [8,9]. Additionally, the COVID-19 pandemic highlighted the importance of standardized and automated reporting systems to ensure timely and accurate data exchange, which is essential for effective public health responses and leads to better health outcomes for patients [4].

The association between public health data integration, automation, and hospital characteristics has become a key focus in assessing reporting system effectiveness, particularly during the COVID-19 pandemic [10]. Beyond improving health care delivery, data integration can enhance hospitals’ operational efficiency, potentially leading to higher profits and increased patient service capacity [11]. However, the most significant barrier to integration remains the lack of standardization in health data norms at local, national, and international levels. Many health data systems cannot communicate effectively, resulting in integration challenges when patients move between health systems [12]. Through improved data integration, public health systems can better address concerns like social determinants of health and disease monitoring for future pandemics while enhancing patient experiences through personalized care. While prior research has examined EHR adoption broadly, few studies have disaggregated public health reporting into its component categories to identify differential adoption patterns across hospital characteristics. This study addresses this gap by simultaneously examining 6 distinct public health reporting categories and analyzing both electronic submission engagement and automation processes as separate outcomes. This context situates the central question of this research: *What hospital characteristics are associated with the adoption and success of automated health reporting systems?* By identifying factors associated with successful implementation of automated health reporting systems, the findings can inform strategies to address disparities and improve public health data infrastructure across different health care settings. This research is particularly significant in light of the COVID-19 pandemic, which exposed weaknesses in current health data systems, especially regarding integration and automation [10]. A well-integrated, automated health data system will not only lead to improved patient outcomes and more patient-focused care but also enhance public health decision-making at both local and national levels [13].

## Methods

### Data Source

The primary data for this study were derived from the 2023 American Hospital Association (AHA) Annual Survey and its supplemental Health Information Technology Survey [14]. The AHA Annual Survey provides comprehensive information on a wide range of hospital characteristics including organizational structure, service lines, staffing, finances, and patient populations. The supplemental Health Information Technology Survey specifically captures detailed information about hospitals’ health information technology capabilities, EHR implementation, and public health reporting practices.

### Outcome Variables

The first set of outcome variables assessed the hospital’s current stage of active engagement towards electronically submitting data for public health reporting across 6 categories: syndromic surveillance, immunization registry, electronic case reporting, public health registry, clinical data registry, and electronic reportable laboratory result reporting. For each category, respondents selected one of five ordinal response options representing implementation stages: (1) actively electronically submitting production data, (2) in the process of testing and validating electronic submission, (3) completed registration to submit data, (4) have not completed registration, or (5) do not know. This variable was operationalized as a dichotomous (yes or no) measure, with “yes” representing hospitals that reported actively electronically submitting production data and those that did not (yes or no). This dichotomization approach was used to create a clear distinction between hospitals actively engaged in electronic reporting versus those at earlier implementation stages or nonparticipants, consistent with prior AHA survey analyses examining health IT adoption [15].

The second set of outcome variables assessed the specific processes used to transmit health data, with respondents identifying whether their hospital utilized automated, manual, or mixed processes across 6 reporting categories. Response options included: (1) fully or primarily automated, (2) mix of automated and manual processes, (3) fully or primarily manual, or (4) do not know. For analysis purposes, the automated reporting variable was operationalized as a binary (yes or no) measure for each of the seven reporting categories, with “yes” representing hospitals using fully or primarily automated processes.

### Confounding Variables

The analysis also included several hospital characteristics and market factors that may influence public health data reporting practices. Hospital ownership type was categorized as government (federal and nonfederal), not-for-profit (private hospitals with Internal Revenue Service 501(c)(3) tax-exempt status), or for-profit (investor-owned facilities operating as taxable business entities). Geographic location was classified as rural or nonrural (urban) based on the hospital’s physical setting and Rural-Urban Commuting Area codes. Hospital size was operationalized using the total staffed bed count and stratified into 3 categories: small (fewer than 100 beds), medium (100‐299 beds), and large (300 or more beds).

System affiliation was measured as a binary variable indicating whether the hospital was part of a larger health care system (system-affiliated) or operated independently. Teaching status was classified using the AHA criteria into nonteaching or teaching. Medicare percentage (proportion of total Medicare inpatient days) and Medicaid percentage (proportion of total Medicaid inpatient visits) were included to account for patient population characteristics that may influence hospitals’ priorities and resource allocation for health IT investments. Market competition was measured using the Herfindahl-Hirschman Index (HHI), calculated based on the distribution of hospital beds within each health care market area. Higher HHI values indicate greater market concentration and less competition, with values approaching 1.0 representing highly concentrated markets [16]. This measure was included to control for the potential influence of competitive pressures on hospitals’ public health reporting practices and technology adoption decisions. These variables were selected based on previous literature identifying them as potential determinants of health care technology adoption, organizational innovation, and public health reporting capabilities.

### Statistical Analysis

This study used descriptive statistics and logistic regression analyses. For categorical variables, we computed frequencies and percentages. For continuous variables (Medicare percentage, Medicaid percentage, and HHI), we calculated means and SDs. We stratified these descriptive statistics by our two primary outcome measures: (1) whether hospitals were actively submitting data electronically and (2) whether hospitals used automated processes for data transmission.

For our primary analysis, we developed a series of multivariable logistic regression models to examine the adjusted associations between hospital characteristics and public health reporting practices. Separate models were constructed for each of the 6 reporting categories (syndromic surveillance, immunization registry, electronic case reporting, public health registry, clinical data registry, and electronic reportable laboratory result reporting) and for both outcome measures (active electronic submission and automated processes).

Results from the logistic regression models are presented as adjusted odds ratios (ORs). We conducted model diagnostics to ensure that all logistic regression assumptions were met. These included tests for multicollinearity using variance inflation factors, examination of influential observations using the Cook distance, and assessment of model fit using the Hosmer-Lemeshow goodness-of-fit test. All analyses were conducted using Stata (version 17.0; StataCorp), with statistical significance set at *P*<.05 for all tests. Cases with missing data on any study variables were excluded from the analysis using listwise deletion.

### Ethical Considerations

In accordance with the policy of the university of North Florida, the Institutional Review Board for the Protection of Human Subjects categorized the research as exempt since the study analyzed secondary data that are publicly available.

## Results

The results reveal patterns in electronic health data reporting practices between health care facilities based on their patient demographics, market concentration measures, and hospital characteristics.

### Actively Submitting Data Electronically

Table 1 reports hospital categorical characteristics across hospitals that actively submit data electronically versus those that do not.

Table 2 reports hospital and market continuous characteristics across hospitals that actively submit data electronically versus those that do not (51.87%-54.14%), while actively submitting facilities demonstrate more consistent Medicare percentages (53.51%-54.13%). The SDs for Medicare percentages are generally higher in nonactive facilities (up to SD 20.15) compared to active facilities (up to SD 16.27). The HHI values for actively submitting facilities (ranging from 0.53 to 0.56, all with SD 0.36) are consistently lower than for nonactive facilities (ranging from 0.59 to 0.67, mostly with SD 0.37). Medicaid percentages are similar between active and nonactive facilities across all reporting categories, with active facilities showing slightly more consistent values (19.28%-20.02%) compared to nonactive facilities (18.6%-20.19%). SDs for Medicaid percentages are also generally higher in nonactive facilities.

**Table 1.** Hospital categorical characteristics across hospitals that actively submit data electronically versus those that do not.

Characteristics	Actively electronically submitting production data (yes or no), n (%)											
	Syndromic surveillance reporting		Immunization registry reporting		Electronic case reporting		Public health registry reporting		Clinical data registry reporting		Electronic reportable laboratory result reporting	
	No	Yes	No	Yes	No	Yes	No	Yes	No	Yes	No	Yes
Ownership												
Government	59 (22.26)	236 (11.77)	36 (16.67)	261 (12.66)	184 (16.08)	111 (9.92)	148 (19.65)	135 (9.15)	170 (17.07)	121 (9.81)	74 (25.61)	219 (11.16)
For-profit	31 (11.7)	287 (14.31)	36 (16.67)	281 (13.63)	139 (12.15)	177 (15.82)	129 (17.13)	184 (12.47)	267 (26.81)	44 (3.57)	38 (13.15)	279 (14.21)
Not-for-profit	175 (66.04)	1482 (73.92)	144 (66.67)	1519 (73.7)	821 (71.77)	831 (74.26)	476 (63.21)	1157 (78.39)	559 (56.12)	1069 (86.63)	177 (61.25)	1465 (74.63)
Rural												
No	131 (49.43)	1295 (64.59)	104 (48.15)	1327 (64.39)	649 (56.73)	775 (69.26)	387 (51.39)	1010 (68.43)	574 (57.63)	828 (67.1)	134 (46.37)	1287 (65.56)
Yes	134 (50.57)	710 (35.41)	112 (51.85)	734 (35.61)	495 (43.27)	344 (30.74)	366 (48.61)	466 (31.57)	422 (42.37)	406 (32.9)	155 (53.63)	676 (34.44)
Size												
Small	167 (63.02)	887 (44.24)	135 (62.5)	923 (44.78)	585 (51.14)	463 (41.38)	437 (58.03)	600 (40.65)	517 (51.91)	512 (41.49)	190 (65.74)	850 (43.3)
Medium	81 (30.57)	819 (40.85)	66 (30.56)	836 (40.56)	425 (37.15)	475 (42.45)	245 (32.54)	637 (43.16)	373 (37.45)	514 (41.65)	78 (26.99)	818 (41.67)
Large	17 (6.42)	299 (14.91)	15 (6.94)	302 (14.65)	134 (11.71)	181 (16.18)	71 (9.43)	239 (16.19)	106 (10.64)	208 (16.86)	21 (7.27)	295 (15.03)
Part of a system												
No	99 (37.36)	377 (18.8)	61 (28.24)	418 (20.28)	303 (26.49)	171 (15.28)	231 (30.68)	238 (16.12)	283 (28.41)	184 (14.91)	115 (39.79)	355 (18.08)
Yes	166 (62.64)	1628 (81.2)	155 (71.76)	1643 (79.72)	841 (73.51)	948 (84.72)	522 (69.32)	1238 (83.88)	713 (71.59)	1050 (85.09)	174 (60.21)	1608 (81.92)
Teaching												
Not teaching	163 (61.51)	905 (45.14)	127 (58.8)	942 (45.71)	582 (50.87)	483 (43.16)	430 (57.1)	621 (42.07)	524 (52.61)	520 (42.14)	180 (62.28)	872 (44.42)
Minor	95 (35.85)	952 (47.48)	82 (37.96)	970 (47.06)	495 (43.27)	547 (48.88)	291 (38.65)	731 (49.53)	442 (44.38)	590 (47.81)	99 (34.26)	947 (48.24)
Major	7 (2.64)	148 (7.38)	7 (3.24)	149 (7.23)	67 (5.86)	89 (7.95)	32 (4.25)	124 (8.4)	30 (3.01)	124 (10.05)	10 (3.46)	144 (7.34)

**Table 2.** Hospital continuous characteristics across hospitals that actively submit data electronically versus those that do not.

Characteristics	Syndromic surveillance (n=2270), mean (SD)	Immunization registry (n=2277), mean (SD)	Electronic case (n=2263), mean (SD)	Public health registry (n=2229), mean (SD)	Clinical data registry (n=2230), mean (SD)	Electronic reportable laboratory results (n=2252), mean (SD)
Not actively electronically submitting production data						
Medicare Percentage	51.87 (20.15)	53.41 (18.56)	54.14 (17.19)	54 (17.99)	53.62 (16.69)	53.97 (18.98)
Medicaid Percentage	20.19 (15.14)	18.6 (12.84)	20.1 (14.23)	19.6 (14.89)	19.29 (14.01)	18.76 (14.66)
Herfindahl-Hirschman Index	0.62 (0.37)	0.62 (0.37)	0.61 (0.37)	0.64 (0.37)	0.59 (0.37)	0.67 (0.36)
Actively electronically submitting production data						
Medicare Percentage	54.13 (15.87)	53.89 (16.26)	53.51 (15.65)	53.9 (15.56)	54.02 (16.27)	53.88 (16.02)
Medicaid Percentage	19.59 (13.15)	19.77 (13.46)	19.28 (12.52)	19.57 (12.37)	20.02 (12.85)	19.84 (13.15)
Herfindahl-Hirschman Index	0.56 (0.36)	0.56 (0.36)	0.53 (0.36)	0.54 (0.36)	0.55 (0.36)	0.55 (0.36)
Total						
Medicare Percentage	53.86 (16.44)	53.85 (16.48)	53.83 (16.44)	53.94 (16.42)	53.84 (16.45)	53.89 (16.43)
Medicaid Percentage	19.66 (13.4)	19.66 (13.4)	19.69 (13.41)	19.58 (13.27)	19.69 (13.38)	19.7 (13.35)
Herfindahl-Hirschman Index	0.57 (0.36)	0.57 (0.36)	0.57 (0.36)	0.57 (0.36)	0.57 (0.36)	0.57 (0.36)

The statistical analysis using logistic regression models is shown in Table 3, which revealed several significant predictors of hospitals’ engagement in electronic health data reporting across different reporting categories. For-profit hospitals show significantly lower odds of engaging in clinical data registry reporting compared to government hospitals (OR 0.15, 95% CI 0.09-0.22*; P*<.001), but higher odds for immunization registry reporting (OR 1.45, 95% CI 1.02-2.07; *P*<.05). Not-for-profit hospitals demonstrate significantly higher engagement in clinical data registry reporting (OR 1.89, 95% CI 1.43-2.50*; P*<.001), electronic case reporting (OR 1.76, 95% CI 1.25-2.48; *P*<.01), and public health registry reporting (OR 1.88, 95% CI 1.41-2.49; *P*<.001) compared to government-owned facilities.

Rural hospitals show significantly reduced likelihood of electronic reporting adoption across immunization registry (OR 0.77, 95% CI 0.61-0.97; *P*<.05), public health registry (OR 0.67, 95% CI 0.46-0.97; *P*<.05), and clinical data registry reporting (OR 0.77, 95% CI 0.60-0.98*; P*<.05) compared to urban counterparts. Hospital size emerges as a significant factor, with medium-sized hospitals showing higher engagement in electronic reportable laboratory results (OR 1.55, 95% CI 1.08-2.22; *P*<.05), public health registry (OR 1.51, 95% CI 1.02-2.25; *P*<.05), clinical data registry (OR 1.35, 95% CI 1.05-1.74; *P*<.05), and syndromic surveillance reporting (OR 1.52, 95% CI 1.06-2.19; *P*<.05) compared to small hospitals. Large hospitals demonstrate even stronger engagement in public health registry (OR 2.13, 95% CI 1.03-4.38*; P*<.05) and syndromic surveillance reporting (OR 2.29, 95% CI 1.17-4.46; *P*<.05).

System affiliation consistently emerges as one of the strongest predictors, with system-affiliated hospitals showing significantly higher odds of electronic reporting engagement across 5 of 6 categories: clinical data registry (OR 2.27, 95% CI 1.80-2.88; *P*<.001), immunization registry (OR 1.70, 95% CI 1.35-2.14; *P*<.001), electronic case reporting (OR 2.16, 95% CI 1.61-2.90; *P*<.001), public health registry (OR 1.78, 95% CI 1.42-2.25; *P*<.001), and electronic reportable laboratory results (OR 1.91, 95% CI 1.41-2.59; *P*<.001). Among teaching status variables, only major teaching hospitals show significantly higher odds for clinical data registry reporting (OR 2.66, 95% CI 1.56-4.52; *P*<.001). Medicare percentage shows a small but significant effect on syndromic surveillance reporting (OR 1.01, 95% CI 1.00-1.02; *P*<.05), while Medicaid percentage shows a minimal significant effect on immunization registry reporting (OR 0.99, 95% CI 0.98-1.00; *P*<.05). These small effect sizes for payer mix variables (ORs close to 1.0) suggest limited practical significance despite statistical significance, likely reflecting the large sample size rather than meaningful clinical impact.

**Table 3.** Logistic regression model of hospitals’ engagement in electronic health data reporting across different reporting categories.

Characteristics	Clinical data registry reporting, OR^a^ (95% CI)	Electronic case reporting, OR (95% CI)	Electronic reportable laboratory result, OR (95% CI)	Immunization registry reporting, OR (95% CI)	Public health registry reporting, OR (95% CI)	Syndromic surveillance reporting, OR (95% CI)
Ownership (reference: government)						
For-profit	0.15^b^ (0.09-0.22)	1.45^c^ (1.02-2.07)	1.38 (0.86-2.22)	0.77 (0.45-1.33)	0.95 (0.66-1.36)	1.45 (0.87-2.42)
Not-for-profit	1.89^b^ (1.43-2.50)	1.31 (0.99-1.72)	1.76^d^ (1.25-2.48)	1.11 (0.72-1.69)	1.88^b^ (1.41-2.49)	1.42 (0.99-2.03)
Rural (reference: no)						
Yes	0.8 (0.62-1.02)	0.77^c^ (0.61-0.97)	0.86 (0.62-1.19)	0.67^c^ (0.46-0.97)	0.77^c^ (0.60-0.98)	0.89 (0.63-1.25)
Size (reference: small)						
Medium	1.22 (0.95-1.58)	1.08 (0.85-1.37)	1.55^c^ (1.08-2.22)	1.51^c^ (1.02-2.25)	1.35^c^ (1.05-1.74)	1.52^c^ (1.06-2.19)
Large	1.12 (0.75-1.67)	1.19 (0.83-1.71)	1.85 (0.97-3.53)	2.13^c^ (1.03-4.38)	1.43 (0.95-2.15)	2.29^c^ (1.17-4.46)
Part of a system (reference: no)						
Yes	2.27^b^ (1.80-2.88)	1.70^b^ (1.35-2.14)	2.16^b^ (1.61-2.90)	1.23 (0.87-1.75)	1.78^b^ (1.42-2.25)	1.91^b^ (1.41-2.59)
Teaching (reference: not teaching)						
Minor teaching	1.04 (0.82-1.30)	1.01 (0.82-1.25)	1.15 (0.84-1.58)	1.07 (0.76-1.52)	1.14 (0.91-1.43)	1.22 (0.88-1.68)
Major teaching	2.66^b^ (1.56-4.52)	1.1 (0.71-1.70)	1.34 (0.58-3.11)	1.26 (0.49-3.28)	1.46 (0.86-2.46)	2.04 (0.81-5.16)
Medicare percentage	1.00 (1.00-1.01)	0.99^c^ (0.98-1.00)	1.00 (0.99-1.01)	1.01 (1.00-1.02)	1.00 (0.99-1.01)	1.01^c^ (1.00-1.02)
Medicaid percentage	1.00 (0.99-1.01)	0.99^d^ (0.98-1.00)	1.00 (0.99-1.02)	1.01 (0.99-1.02)	0.99 (0.98-1.00)	1.00 (0.99-1.01)
Herfindahl-Hirschman Index	0.99 (0.72-1.36)	0.82 (0.61-1.10)	0.74 (0.48-1.14)	1.17 (0.73-1.88)	0.76 (0.56-1.04)	1.17 (0.76-1.81)

a OR: odds ratio.

b *P*<.001.

c *P*<.05.

d *P*<.01.

### Automated Processes to Transmit Public Health Data

Table 4 reports hospital categorical characteristics across hospitals that have automated processes to transmit public health data (71.55%‐87.03% of “yes” responses), with particularly strong adoption for clinical data registry reporting (87.03%). Government hospitals show the lowest representation among automated reporting adopters (8.10%‐11.45%), while for-profit hospitals show moderate adoption that varies by reporting type, with notably higher representation in electronic case reporting (20.35%). The rural-urban divide is substantial, with nonrural hospitals constituting the clear majority of facilities using automated processes across all reporting categories (64.83%‐71.72%). The imbalance is most pronounced for clinical data registry reporting, where rural hospitals represent only 28.28% of automated adopters despite making up 40.86% of facilities not using automation for this purpose.

Hospital size shows a clear pattern where larger hospitals are disproportionately represented among automated process adopters. Medium and large hospitals together represent 55% to 60% of facilities using automation across reporting categories, despite making up only 40% to 47% of nonautomated facilities. Small hospitals, while still numerous among automation adopters (38.6%‐43.77%), are significantly under-represented compared to their share among nonautomated facilities (49.18%‐67.37%). System affiliation emerges as one of the strongest predictors, with system-affiliated hospitals representing 81.22% to 86% of facilities using automated processes across reporting categories. This contrasts sharply with their 62.84% to 76.13% representation among nonautomated facilities. Finally, teaching status also shows consistent patterns, with minor teaching and major teaching hospitals combined representing 51.25% to 60.54% of automated adopters across reporting categories, compared to 38.49% to 48.17% of nonautomated facilities. Major teaching hospitals, despite their small numbers overall, show consistently higher representation among automated facilities (6.63%‐10.5%) compared to nonautomated ones (3.63%‐10.5%).

**Table 4.** Hospital categorical characteristics across hospitals that have automated processes to transmit public health data versus those that do not.

Characteristics	Automated processes to transmit the data (yes or no), n (%)											
	Syndromic surveillance reporting		Immunization registry reporting		Electronic case reporting		Public health registry reporting		Clinical data registry reporting		Electronic reportable laboratory result reporting	
	No	Yes	No	Yes	No	Yes	No	Yes	No	Yes	No	Yes
Ownership												
Government	85 (20.38)	207 (11.27)	75 (22.66)	217 (11.45)	197 (18.11)	88 (8.1)	199 (18.27)	83 (8.41)	221 (15.84)	60 (8.75)	120 (23.35)	167 (10.07)
For-profit	36 (8.63)	281 (15.3)	51 (15.41)	245 (12.93)	72 (6.62)	221 (20.35)	69 (6.34)	158 (16.01)	198 (14.19)	29 (4.23)	54 (10.51)	197 (11.87)
Not-for-profit	296 (70.98)	1349 (73.43)	205 (61.93)	1433 (75.62)	819 (75.28)	777 (71.55)	821 (75.39)	746 (75.58)	976 (69.96)	597 (87.03)	340 (66.15)	1295 (78.06)
Rural												
No	228 (54.68)	1191 (64.83)	152 (45.92)	1237 (65.28)	611 (56.16)	752 (69.24)	627 (57.58)	686 (69.5)	825 (59.14)	492 (71.72)	265 (51.56)	1119 (67.45)
Yes	189 (45.32)	646 (35.17)	179 (54.08)	658 (34.72)	477 (43.84)	334 (30.76)	462 (42.42)	301 (30.5)	570 (40.86)	194 (28.28)	249 (48.44)	540 (32.55)
Size												
Small	240 (57.55)	804 (43.77)	223 (67.37)	819 (43.22)	582 (53.49)	429 (39.5)	579 (53.17)	381 (38.6)	686 (49.18)	275 (40.09)	302 (58.75)	700 (42.19)
Medium	133 (31.89)	765 (41.64)	83 (25.08)	788 (41.58)	355 (32.63)	500 (46.04)	363 (33.33)	447 (45.29)	515 (36.92)	300 (43.73)	153 (29.77)	706 (42.56)
Large	44 (10.55)	268 (14.59)	25 (7.55)	288 (15.2)	151 (13.88)	157 (14.46)	147 (13.5)	159 (16.11)	194 (13.91)	111 (16.18)	59 (11.48)	253 (15.25)
Part of a system												
No	125 (29.98)	345 (18.78)	123 (37.16)	351 (18.52)	301 (27.67)	152 (14)	299 (27.46)	154 (15.6)	333 (23.87)	117 (17.06)	171 (33.27)	295 (17.78)
Yes	292 (70.02)	1492 (81.22)	208 (62.84)	1544 (81.48)	787 (72.33)	934 (86)	790 (72.54)	833 (84.4)	1062 (76.13)	569 (82.94)	343 (66.73)	1364 (82.22)
Teaching												
Not teaching	237 (56.83)	822 (44.75)	211 (63.75)	834 (44.01)	564 (51.84)	454 (41.8)	562 (51.61)	401 (40.63)	690 (49.46)	277 (40.38)	295 (57.39)	720 (43.4)
Minor	155 (37.17)	885 (48.18)	108 (32.63)	918 (48.44)	444 (40.81)	560 (51.57)	450 (41.32)	511 (51.77)	626 (44.87)	337 (49.13)	184 (35.8)	821 (49.49)
Major	25 (6)	130 (7.08)	12 (3.63)	143 (7.55)	80 (7.35)	72 (6.63)	77 (7.07)	75 (7.6)	79 (5.66)	72 (10.5)	35 (6.81)	118 (7.11)

Table 5 reports hospital and market continuous characteristics across hospitals that have automated processes to transmit public health data versus those that do not.

The logistic regression analysis examined factors associated with hospitals’ use of automated processes (EHR-generated data sent electronically or automatically) to transmit data to public health agencies across 6 reporting categories (Table 6). Hospital ownership was shown to significantly impact automated reporting practices. For-profit hospitals are 85% less likely than government hospitals to use automated processes for clinical data registry reporting (OR 0.15, 95% CI 0.09-0.22; *P*<.001), but 45% more likely to automate immunization registry reporting (OR 1.45, 95% CI 1.02-2.07; *P*<.05). Not-for-profit hospitals show significantly higher automation adoption in clinical data registry reporting (OR 1.89, 95% CI 1.43-2.50; *P*<.001), electronic case reporting (OR 1.76, 95% CI 1.25-2.48; *P*<.01), and public health registry reporting (OR 1.88, 95% CI 1.41-2.49; *P*<.001) compared to government facilities.

Rural status negatively impacts automation adoption, with rural hospitals showing significantly lower odds of automated data transmission for immunization registries (OR 0.77, 95% CI0.61-0.97; *P*<.05), public health registries (OR 0.67, 95% CI 0.46-0.97; *P*<.05), and clinical data registries (OR 0.77, 95% CI 0.60-0.98; *P*<.05). Hospital size matters, with medium-sized hospitals showing higher odds of automation across electronic reportable laboratory results (OR 1.55, 95% CI 1.08-2.22; *P*<.05), immunization registries (OR 1.51, 95% CI 1.02-2.25; *P*<.05), public health registries (OR 1.35, 95% CI 1.05-1.74; *P*<.05), and syndromic surveillance (OR 1.52, 95% CI 1.06-2.19; *P*<.05) compared to small hospitals. Large hospitals show even stronger automation adoption in immunization registries (OR 2.13, 95% CI 1.03-4.38; *P*<.05) and syndromic surveillance (OR 2.29, 95% CI 1.17-4.46; *P*<.05).

System affiliation emerges as the most consistent predictor of automation adoption, with system-affiliated hospitals showing significantly higher odds of automated reporting across 5 categories: clinical data registry (OR 2.27, 95% CI 1.80-2.88; *P*<.001), immunization registry (OR 1.70, 95% CI 1.35-2.14; *P*<.001), electronic case reporting (OR 2.16, 95% CI 1.61-2.90; *P*<.001), public health registry (OR 1.78, 95% CI 1.42-2.25; *P*<.001), and electronic reportable laboratory results (OR 1.91, 95% CI 1.41-2.59; *P*<.001). Major teaching status significantly increases automation adoption for clinical data registry reporting (OR 2.66, 95% CI 1.56-4.52; *P*<.001), while Medicare and Medicaid percentages show minimal but significant effects on syndromic surveillance and immunization registry reporting, respectively. Market concentration (HHI) shows no significant association with automation adoption across all reporting categories.

**Table 5.** Hospital continuous characteristics across hospitals that have automated processes to transmit public health data versus those that do not.

Characteristics	Syndromic surveillance (n=2254), mean (SD)	Immunization registry (n=2226), mean (SD)	Electronic case (n=2174), mean (SD)	Public health registry (n=2076), mean (SD)	Clinical data registry (n=2081), mean (SD)	Electronic reportable laboratory results (n=2173), mean (SD)
No automated processes to transmit the data						
Medicare Percentage	52.69 (19.31)	54.51 (19.31)	53.79 (18.02)	53.77 (17.71)	53.41 (17.26)	53.95 (19.03)
Medicaid Percentage	20.29 (15.27)	18.24 (14.59)	19.69 (14.45)	19.56 (14.42)	19.87 (13.98)	19.1 (14.57)
Herfindahl-Hirschman Index	0.6 (0.36)	0.64 (0.35)	0.59 (0.36)	0.59 (0.36)	0.59 (0.36)	0.61 (0.36)
Automated processes to transmit the data						
Medicare Percentage	54.23 (15.69)	53.83 (16.04)	54.04 (14.89)	53.68 (15.24)	54.57 (15.28)	53.75 (15.69)
Medicaid Percentage	19.43 (12.85)	19.8 (13.15)	19.33 (12.1)	19.65 (12.04)	19.13 (12.08)	19.83 (12.96)
Herfindahl-Hirschman Index	0.56 (0.36)	0.56 (0.36)	0.56 (0.36)	0.53 (0.36)	0.52 (0.36)	0.55 (0.36)
Total						
Medicare Percentage	53.95 (16.43)	53.93 (16.56)	53.92 (16.52)	53.73 (16.58)	53.79 (16.64)	53.8 (16.54)
Medicaid Percentage	19.59 (13.33)	19.57 (13.39)	19.51 (13.33)	19.6 (13.34)	19.63 (13.39)	19.66 (13.36)
Herfindahl-Hirschman Index	0.57 (0.36)	0.58 (0.36)	0.57 (0.36)	0.56 (0.36)	0.57 (0.36)	0.56 (0.36)

**Table 6.** Logistic regression analysis of factors associated with hospitals’ use of automated processes (electronic health record [EHR]-generated data sent electronically or automatically) to transmit data to public health agencies across 6 reporting categories.

Characteristics	Clinical data registry reporting, OR^a^ (95% CI)		Electronic case reporting, OR (95% CI)		Electronic reportable laboratory result, OR (95% CI)		Immunization registry reporting, OR (95% CI)		Public health registry reporting, OR (95% CI)		Syndromic surveillance reporting, OR (95% CI)	
Ownership (reference: government)												
For-profit	0.15^b^ (0.09-0.22)		1.45^c^ (1.02-2.07)		1.38 (0.86-2.22)		0.77 (0.45-1.33)		0.95 (0.66-1.36)		1.45 (0.87-2.42)	
Not-for-profit	1.89^b^ (1.43-2.50)		1.31 (0.99-1.72)		1.76^d^ (1.25-2.48)		1.11 (0.72-1.69)		1.88^b^ (1.41-2.49)		1.42 (0.99-2.03)	
Rural (reference: no)												
Yes	0.8 (0.62-1.02)		0.77^c^ (0.61-0.97)		0.86 (0.62-1.19)		0.67^c^ (0.46-0.97)		0.77^c^ (0.60-0.98)		0.89 (0.63-1.25)	
Size (reference: small)												
Medium	1.22 (0.95-1.58)		1.08 (0.85-1.37)		1.55^c^ (1.08-2.22)		1.51^c^ (1.02-2.25)		1.35^c^ (1.05-1.74)		1.52^c^ (1.06-2.19)	
Large	1.12 (0.75-1.67)		1.19 (0.83-1.71)		1.85 (0.97-3.53)		2.13^c^ (1.03-4.38)		1.43 (0.95-2.15)		2.29^c^ (1.17-4.46)	
Part of a system (reference: no)												
Yes	2.27^b^ (1.80-2.88)		1.70^b^ (1.35-2.14)		2.16^b^ (1.61-2.90)		1.23 (0.87-1.75)		1.78^b^ (1.42-2.25)		1.91^b^ (1.41-2.59)	
Teaching (reference: not teaching)												
Minor teaching	1.04 (0.82-1.30)		1.01 (0.82-1.25)		1.15 (0.84-1.58)		1.07 (0.76-1.52)		1.14 (0.91-1.43)		1.22 (0.88-1.68)	
Major teaching	2.66^b^ (1.56-4.52)		1.1 (0.71-1.70)		1.34 (0.58-3.11)		1.26 (0.49-3.28)		1.46 (0.86-2.46)		2.04 (0.81-5.16)	
Medicare Percentage	1.00 (1.00-1.01)		0.99^c^ (0.98-1.00)		1.00 (0.99-1.01)		1.01 (1.00-1.02)		1.00 (0.99-1.01)		1.01^c^ (1.00-1.02)	
Medicaid Percentage	1.00 (0.99-1.01)		0.99^d^ (0.98-1.00)		1.00 (0.99-1.02)		1.01 (0.99-1.02)		0.99 (0.98-1.00)		1.00 (0.99-1.01)	
Herfindahl-Hirschman Index	0.99 (0.72-1.36)		0.82 (0.61-1.10)		0.74 (0.48-1.14)		1.17 (0.73-1.88)		0.76 (0.56-1.04)		1.17 (0.76-1.81)	

a OR: odds ratio.

b *P*<.001.

c *P*<.05.

d *P*<.01.

## Discussion

### Principal Findings

This study identifies some of the main differences in automation and integrating public health information between US hospitals driven by structural resource inequalities, institutional practice, and location. Rural, independent, and smaller hospitals lag far behind urban, system-affiliated, and larger hospitals when it comes to adopting automated reporting systems. Despite national-level attempts to standardize health IT infrastructure, these gaps underscore systemic obstacles based on financial interests, organizational capacities, and market forces.

Rural hospitals continue to face significant challenges in adopting electronic public health reporting despite national progress in health IT adoption [15]. Limited financial resources and constrained operational capacity hinder their ability to invest in the infrastructure required for automation. These facilities often serve smaller patient populations and receive lower reimbursement rates, which makes it difficult to justify the high upfront costs of implementing advanced reporting systems. Additionally, rural hospitals typically lack access to IT specialists and foundational systems that support seamless electronic data exchange, resulting in a greater reliance on manual or mixed reporting methods. These barriers not only restrict their compliance with public health reporting requirements but also widen the digital divide between rural and urban health care providers. Addressing these disparities requires targeted policy support and financial investment to ensure rural hospitals can fully participate in the public health data ecosystem.

The nonrural versus rural divide is stark in the results as both the rates of actively submitting data electronically and the adoption of automated processes to transmit that public health data show low rates of submission and adoption by rural hospitals in all reporting categories. There are many possible reasons for this difference largely relating to the differing economic environments of these hospitals. Rural hospitals often face greater financial strain due to the poorer socioeconomic conditions of their locals and thus do not have the financial capital to invest in high-tech systems. As Younis [17] shared that rural hospitals generate less revenue than urban hospitals and are significantly disadvantaged in terms of performance.

Another avenue to look at is the role of competition from other hospitals that nonrural hospitals face. As discussed in Garcia-Lacalle and Martin [18], hospitals in a market-driven environment have a keen sense of where they sit in comparison to their competition and therefore consider new strategies to better focus on patients and users. Once one hospital in a competitive environment adopts an electronic data submission system or automates their pre-existing one, it encourages other hospitals in that same environment to also adopt. Similarly, Ghiasi et al [19] found, in their literature review, that hospitals in a competitive market seek to differentiate themselves from competitors through specific services. Some of these differentiating services could be electronic data submission systems.

In our study, larger hospitals benefit from centralized IT infrastructure and specialized personnel, enabling consistent compliance with evolving standards. Medium and large hospitals show 1.5 to 2.3 times higher odds of automation across categories like syndromic surveillance and laboratory reporting. These institutions absorb upfront costs more effectively and maintain robust EHR systems, whereas smaller facilities struggle with limited staffing and budgetary flexibility. Particularly larger hospitals within multihospital systems demonstrate higher engagement in both active electronic data submission and automated reporting due to greater resource availability. These hospitals benefit from economies of scale that support investment in centralized IT infrastructures and EHR systems. In addition, system-affiliated hospitals are also more likely to have internal health IT teams and established workflows for public health communication because it reduces barriers to implementation.

Not-for-profit hospitals lead in adoption due to mission-driven commitments to population health and access to grant funding. Their focus on community benefit aligns with public health reporting goals, whereas for-profit hospitals prioritize revenue-generating technologies (eg, billing systems). Government hospitals, with the limitations of bureaucratic procurement systems, fall behind despite regulatory encouragement. The trends are indicative of findings by Tsai et al [20] that financial restrictions and fragmented workflows are the main barriers against EHR adoption in low-resource settings.

In our study, facilities not actively submitting data electronically exhibit more variable Medicare percentages (51.87%‐54.14%), suggesting that markets with less competition (higher HHI values) reduce pressure to adopt reporting technologies. Lower digital literacy among older Medicare populations may also deprioritize automation in regions serving these demographics. Conversely, hospitals in competitive, high-volume markets align IT investments with performance metrics to meet patient and regulatory expectations.

### Policy Implications

A 2024 analysis by the Kaiser Family Foundation found that nearly half of US metropolitan areas are dominated by just one or two hospital systems, significantly reducing competition and, consequently, the urgency for these institutions to adopt advanced data reporting practices [21]. This aligns with findings from the BMC Health Services Research, which revealed that providers in rural or less competitive regions demonstrate lower EHR adoption and interoperability [22]. Moreover, patient population characteristics, particularly among older adults on Medicare, further influence reporting engagement. A systematic review in the Archives of Public Health emphasized the digital health literacy gap in this group, suggesting that facilities serving older or underserved populations may deprioritize electronic data initiatives due to lower patient engagement with digital platforms [23]. These studies underscore the multifactorial barriers to robust public health data reporting, suggesting the need for targeted policy incentives and infrastructure support to promote broader and more equitable adoption.

Limitations

This study’s limitation lies in its reliance on secondary data from the 2023 AHA Annual Survey. Hospital characteristics are based on self-reported data which may affect accuracy. Our cross-sectional design limits causal inference. This analysis focused on US hospitals only, affecting generalizability to other types of health organizations and countries. Finally, the rural or urban classification using Rural-Urban Commuting Area codes may not fully capture rural-urban distinctions. Residual confounding may exist due to unmeasured variables such as IT staffing levels or leadership engagement.

### Conclusions

The clear difference between nonrural and rural hospitals in terms of electronic data submission and automation adoption shows significant gaps caused by economic and competitive factors. Nonrural hospitals, benefiting from higher revenue and competitive pressures, are more likely to invest in advanced IT systems and automated processes. On the other hand, rural hospitals face financial constraints and lower patient volumes, limiting their ability to adopt such technologies. This divide is further worsened by the centralized resource allocation and organized workflows in system-aligned hospitals, which improve their reporting capabilities. Not-for-profit hospitals also lead in electronic health data adoption due to their mission-driven priorities and access to grant funding. Research highlights the many barriers to strong public health data reporting, shaped by market dynamics and patient demographics. Effective strategies for improving electronic data submission may include tailored incentives, strategic partnerships, and population-specific approaches. Addressing these gaps is crucial for ensuring fair access to advanced health care technologies and improving overall public health reporting. Targeted policy interventions and financial support are essential to help rural hospitals overcome structural barriers and participate more fully in the nation’s public health data system.

## Abbreviations

AHA: American Hospital Association
AI: artificial intelligence
EHR: electronic health record
HHI: Herfindahl-Hirschman Index
OR: odds ratio
